# SMT-based verification of program changes through summary repair

**DOI:** 10.1007/s10703-023-00423-0

**Published:** 2023-05-04

**Authors:** Sepideh Asadi, Martin Blicha, Antti E. J. Hyvärinen, Grigory Fedyukovich, Natasha Sharygina

**Affiliations:** 1 Università della Svizzera italiana, Lugano, Switzerland; 2 Florida State University, Tallahassee, FL USA; 3 Charles University, Prague, Czech Republic

**Keywords:** Symbolic model checking, Incremental verification, SMT solving, Craig interpolation, Program changes

## Abstract

This article provides an innovative approach for verification by model checking of programs that undergo continuous changes. To tackle the problem of repeating the entire model checking for each new version of the program, our approach verifies programs incrementally. It reuses computational history of the previous program version, namely function summaries. In particular, the summaries are over-approximations of the bounded program behaviors. Whenever reusing of summaries is not possible straight away, our algorithm repairs the summaries to maximize the chance of reusability of them for subsequent runs. We base our approach on satisfiability modulo theories (SMT) to take full advantage of lightweight modeling approach and at the same time the ability to provide concise function summarization. Our approach leverages pre-computed function summaries in SMT to localize the checks of changed functions. Furthermore, to exploit the trade-off between precision and performance, our approach relies on the use of an SMT solver, not only for underlying reasoning, but also for program modeling and the adjustment of its precision. On the benchmark suite of primarily Linux device drivers versions, we demonstrate that our algorithm achieves an order of magnitude speedup compared to prior approaches.

## Introduction

Modern software is developed by multitudes of developers from all over the world. As a result, the software undergoes frequent minor changes, e.g., bug fixes, introduction of new features, optimizations, refactoring, and so on. The problem of updating software is that this might break existing features—bugs might get introduced. The confidence of correctness can be increased by rigorous verification before a new revision of a piece of software is checked in. Model checking techniques [1, 2] verify program fully automatically and exhaustively. However, most software verification approaches are not designed to support sequences of program versions, and they force each changed program to be verified from scratch which often makes re-verification computationally impractical.

This work addresses the problem of efficient analysis of a program after it undergoes changes. As a viable solution to this problem, incremental verification is a promising approach that aims to *reuse* the invested efforts between verification runs. We base our approach on symbolic verification techniques, and in particular bounded model checking (BMC) [3]. The previous work [4] has shown that the idea of extracting and reusing *function summaries* across program changes is useful in BMC when using the so called bit-blasting approach, with the direct use of a SAT solver. However, due to the known complexity of bit-precise encoding it suffers from scalability issues. Using SAT-based function summaries also creates summaries that can be significantly larger than the original formulas, and that are not human-readable, impeding their reuse and maintainability.

We present an incremental BMC approach that is based on Satisfiability Modulo Theories (SMT) [5]. Since SMT formulas provide a more natural and lightweight representation than purely propositional logic [6], it allows for a more succinct summary representation in first-order logic. We tackle scalability issues that arise due to verification of industrial-size program versions by (i) modeling the program with fragments of quantifier-free first-order logic, in particular in Linear Real Arithmetic ($\textrm{LRA}$) and Equality with Uninterpreted Functions ($\textrm{EUF}$) which allows to leverage success of nowadays SMT solvers, and (ii) reusing and maintaining *SMT-based function summaries* across the closely-related programs.

Function summaries are obtained from Craig Interpolants [7] which play the role of conserved function specifications. For a code that recurs in several versions, function summaries have been proven successful as a means to capture the properties relevant for BMC in a form that avoids duplicate verification [4, 8–13]. Function summaries constructed in SMT are more succinct and thus their easier reuse. In this article, we exploit an SMT-based family of summaries that condenses the relevant information from a previous verification run to localize and speed up the checks of new program versions. In this approach, the problem of determining whether a newly changed program still meets a safety property reduces to the problem of validating the family of summaries for the new program. As a result, in practice the verification stays often very local, resulting in significant run time improvements.

Our proposed *incremental BMC* solution aims to maintain and repair over-approximating summaries of all the program functions. Overall, our solution proceeds as follows. First, a program together with safety properties is modeled in SMT, and if properties hold, function summaries are computed. Then once a change arrives, it first determines whether the old summaries for functions are still valid after the change. This validation phase is local to the change and tends to be computationally inexpensive since it considers only the changed function bodies, their old summaries, and possibly the summaries of the predecessors of the changed functions. If this local validation phase succeeds, the new program version is also safe. If local validation fails, the approach attempts to widen the scope of the search while still maintaining some locality, by propagating the validation check to the callers of the modified functions. After each successful validation, any invalidated summaries become a candidate to be repaired and are made available for checking the next program version.

The key idea behind the *summary repair* approach is to circumvent the deletion of invalidated function summaries and instead attempt to adapt them to the changed functions. Our solution repairs an over-approximating summary of the program function which is coarse enough to enable rapid check but strong enough to cover more changes in an incremental checking scenario. The repair is done via two strategies: the first *weakens* the invalid summary formulas by removing the broken parts, and the second *strengthens* the weakened summary by recomputing the corresponding interpolant and adding missing parts. We keep the conjunction of newly repaired summaries for subsequent uses. The refinement procedure accompanies our incremental summary validation algorithm for dealing with spurious behaviors that might be introduced due to imprecise over-approximative summaries.

We implemented our SMT-based incremental verification algorithm with the new concept of summary repair in the UpProver tool. We advocate the necessity to offer various encoding options to the user. Therefore, in addition to the provided SMT-level light-weight modeling and the corresponding SMT-level summarizations supported by our incremental verifier, the tool allows adjusting the precision and efficiency with different levels of encodings. For this purpose UpProver enables the $\textrm{LRA}$ and $\textrm{EUF}$ theories (and in the future, more). This was not possible in the previous-generation tools based on bit blasting (e.g., in its predecessor eVolCheck [4, 12]), and this distinguishes UpProver from them. Furthermore, our approach not only allows the reuse of summaries obtained from SMT-based interpolation, but also provides an innovative capability of repairing them automatically and using them in the subsequent verification runs.

*Improvement over previous own work. * The present work is an extension of [14] published in a conference. We build upon and extend [14] in a number of ways: (i) we provide a unified and richer description of the SMT-based verification framework for program revisions; (ii) we propose a new algorithm to repair previously computed summaries on-the-fly and to use them in the subsequent verification runs; (iii) we give an algorithm for constructing formulas in the presence of various substitution scenarios and refining the over-approximation that are not accurate enough; (iv) we provide a proof of correctness of the algorithm and then discusses how it can be instantiated to a concrete theory of SMT with different interpolation procedures; (v) a thorough evaluation of the proposed algorithm is carried out on industrial verification problems created from Linux kernel device drivers with several revisions.

*Structure of the paper. * Sect. 2 provides the necessary background used in the theoretical development of the work. Section 3 uses a concrete example of a two-version model checking problem to demonstrate how the approach can incrementally verify the second program by reusing pre-computed summaries of the first program. Section 4 first presents the core algorithm for incremental verification of program revisions, then introduces the algorithm for summary repair, and lastly describes an improvement for efficiently building formulas and refining them on-the-fly. Section 5 first discusses the correctness of our proposed algorithm and then instantiates the algorithm with respect to a concrete theory. Section 6 gives an overview of the architecture and implementation of our tool, UpProver. Section 7 describes the experimental results and evaluation of our tool. We discuss related work in Sect. 8 and finally conclude in Sect. 9.

## Background

We first review symbolic program modeling and interpolation-based function summarization. Then we describe how to compute function summaries and how to apply them while encoding a program.

### Program modeling

We work in the domain of Satisfiability Modulo Theories (SMT) [5] where satisfiability of formulas is determined with respect to some background theory $\mathcal {T}$. In this work, we restrict our interest to quantifier-free formulas. Since our goal is an efficient analysis of a program after a change, to leverage the success of nowadays SMT solvers we concentrate on two light-weight theories of SMT for program encodings: (i) the theory of equality with uninterpreted functions ($\textrm{EUF}$), and (ii) the theory of linear real arithmetic ($\textrm{LRA}$). However, our approach is generic and is not bound to specific theories.

Bounded Model Checking (BMC) [3] is one of the widely-used bug-catching techniques that trades off completeness of the state-space exploration for finding as many counter-examples as allowed by the predetermined time- and resource constraints. SMT-based BMC has been successfully applied in verifying standalone programs [11, 15, 16]. Our SMT-based BMC technique aims at verifying different program versions and operates on a loop-free program created from an original by unrolling all loops and recursive calls up to a given number of unwinding steps.

We write loop-free programs as tuples $$P = (F, f_{ main })$$ where *F* represents the finite set of unique function calls, i.e., function invocation with a unique combination of a program location, a call stack, and a target function. $$f_{ main } \in F$$ denotes the call of the entry point of the program. Interchangeably *F* also corresponds to the set of functions in the call tree of the unrolled program. We use relations $$ child \subseteq F \times F$$ and $$ subtree \subseteq F \times F$$, where $$ child $$ relates each function *f* to all the functions invoked by *f*, and $$ subtree $$ is a reflexive transitive closure of *child*. Since each node has at most one parent, we write $$ parent (n_2)$$ to refer to $$n_1$$ if $$ child (n_1, n_2)$$ holds.

Classical BMC of software encodes an unwound program to a BMC formula [17]. First, the unrolled program is encoded into the static single assignment form (SSA), from which a BMC formula is constructed. However the resulting formula is monolithic and all the function calls are inlined, thus it does not allow modular verification. Let ${\beta _{f}}$ be the BMC encoding of the body of a function *f*, i.e., the logical formula obtained from the SSA form of the body of the function *f*. Note that ${\beta _{f}}$ does not include inlining of called functions. A *partitioned BMC* (PBMC) formula [8] is constructed recursively as

1 $$\begin{aligned} {\texttt {InlineFormula}}({f}) \triangleq {\beta _{f}} \wedge \bigwedge _{{h} \in {F}: child({f}, {h})}{\texttt {InlineFormula}}({h}) \end{aligned}$$

For each ${f} \in {F}$, the formula is built by conjoining the partition ${\beta _{f}}$ and a separate partition for all nested calls. The PBMC formula ${\texttt {InlineFormula}}({f}_{ main )}$ conjoined with the negation of a safety property $error_{f_{main}}$ is called a *safety query*. Typically $error_{f_{main}}$ represents disjunction of the negations of each of the assertion in the program. A program is *safe* if the safety query is unsatisfiable.

#### Interpolation-based function summarization

Function summary is an over-approximation of the function behavior defined as a relation over its input and output variables. There are different ways to obtain a summary formula, and in this work we use the approach introduced in [8] for extracting summary formulas using Craig interpolation. Craig interpolation [7] is widely used as a means of abstraction in symbolic model checking [18, 19]. Interpolants can be computed from a proof of unsatisfiability of the formula $A \wedge B$.

##### Definition 1

(binary interpolation) Given an unsatisfiable formula $\Phi$ partitioned into two disjoint formulas *A* and *B*, we call the pair $(A \mid B)$ a *binary interpolation instance*. An *interpolation algorithm* $Itp$ is a procedure that maps an interpolation instance to a formula $I = Itp (A \mid B)$ such that (i) $A \Rightarrow I$, (ii) $I \wedge B \Rightarrow \bot$, and (iii) *I* is defined over symbols appearing both in *A* and *B*.

Binary interpolation can be generalized so that the partitions of an unsatisfiable formula form a tree structure. We in particular concentrate on *tree interpolants*, generalizations of binary interpolants, obtained from a single proof that guarantees the tree interpolation property as defined in the following:

##### Definition 2

(tree interpolation property.^1^) Let $X_1 \wedge \ldots \wedge X_n \wedge Y \wedge Z$ be an unsatisfiable formula in first-order logic. Let $I_{X_1}, \ldots , I_{X_n}$ and $I_{X_1 \ldots X_n Y}$ be interpolants for interpolation instances $(X_1 \mid X_2 \wedge \ldots \wedge X_n \wedge Y \wedge Z)$, $\ldots$, $(X_n \mid X_1 \wedge \ldots \wedge X_{n-1} \wedge Y \wedge Z)$, and $(X_1 \wedge \ldots \wedge X_n \wedge Y \mid Z)$, respectively. The tuple $(I_{X_1}, \ldots , I_{X_n}, Y,I_{X_1\ldots X_n Y})$ has the *tree interpolation property* iff $I_{X_1} \wedge \ldots \wedge I_{X_n} \wedge Y \Rightarrow I_{X_1 \ldots X_n Y}$.

Note that an interpolation procedure that guarantees tree interpolation property according to Definition 2 also computes a tree interpolant for a tree interpolation problem as defined in [21]. The properties of tree interpolant follow from Definition 2 when for each node in the tree interpolation problem, the following partitioning of the formula is considered: the subtrees of the current node’s children are the partitions $X_1,\ldots ,X_n$, the label of the current node is the partition *Y*, and the rest of the formula forms the partition *Z*.

In the following, we show how interpolation can be used to extract over-approximation of the function behaviors after a successful verification run. By exploiting the proof of unsatisfiability for the safety query we can extract *function summaries* [8] for each function call *f*.
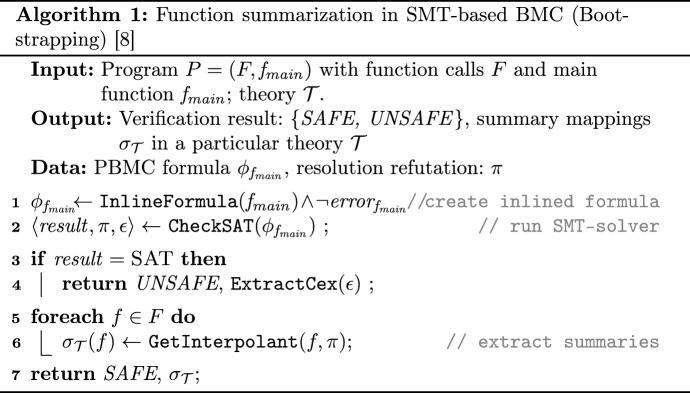


Algorithm 1 describes the method for constructing function summaries in BMC. At line 1 once the safety query in a certain theory $\mathcal {T}$ is created, it is sent to an SMT solver. If the result is unsatisfiable, i.e., the program is safe, the method $\texttt {GetInterpolant}$ (line 6) computes an interpolant for each $f \in F$ from the proof of unsatisfiability. Function summaries for a function *f* are constructed as interpolants as follows: The PBMC formula $\Phi$ is divided into two parts ${\varPhi ^{subtree}_{f}} \wedge {\varPhi ^{rest}_{f}}$. First, ${\varPhi ^{subtree}_{f}}$ corresponds to the partitions representing the function call *f* and its nested function calls:

2 $$\begin{aligned} {\varPhi ^{subtree}_{f}} \triangleq \bigwedge _{h \in {F} : subtree(f, h)} {\beta _{h}} \end{aligned}$$

Second, ${\varPhi ^{rest}_{f}}$ corresponds to the rest of the program including the negation of safety properties:

3 $$\begin{aligned} {\varPhi ^{rest}_{f}} \triangleq \lnot error_{f_{main}} \wedge \bigwedge _{h \in {F} : \lnot subtree({f}, {h})} {\beta _{h}} \end{aligned}$$

Then for each *f*, formula (2) is considered as *A*-part and formula (3) as *B*-part. The $\texttt {GetInterpolant}$ method generates an interpolant $I_{f}$ for the interpolation instance $(A \mid B)$, which acts as a summary for the function *f*. We map functions to their summaries encoded in the theory $\mathcal {T}$ with $\sigma _{\mathcal {T}}$: ${F} \rightarrow {S}$ such that $\sigma _{\mathcal {T}}$ $({f}) = I_{{f}}$.

It is important to note that although the bootstrapping phase can take time, especially if the program being bootstrapped is large, it is a critical part of the approach as it can save significant resources in subsequent runs. If a program has only one function and no function calls, no function summary will be generated. This means that the algorithm will function similarly to a classical non-incremental BMC, and the efficiency of our algorithm may be limited in this scenario.

### Applying summaries in formula construction


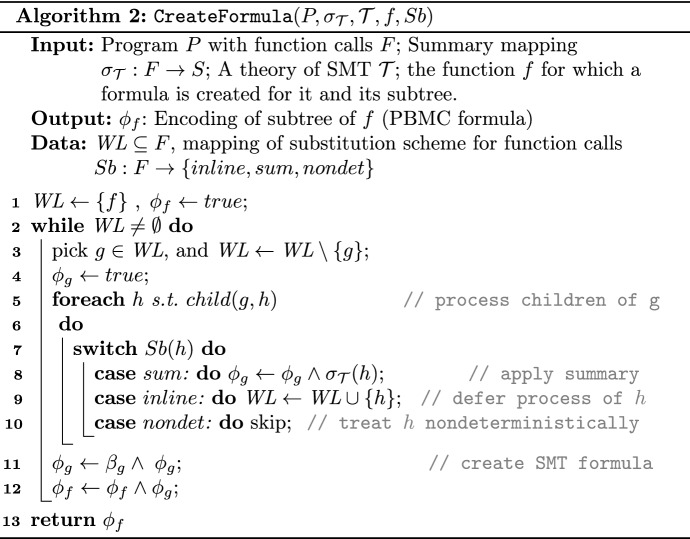


Construction of the formulas by inlining as in Eq. (1) results in a monolithic formula where the whole function bodies are included. We describe the construction of PBMC formula in the presence of function summaries that can be substantially smaller compared to the inlining of the entire function bodies since functions summaries tend to be more compact.

Consider function *f* as a root of a subtree of a program. Suppose in the subtree of *f* there is a function *h* and its summary was already computed. Then the summary of *h* can be substituted for body of *h* while building the PBMC formula of *f*. This way, the PBMC formula $\phi _{f}$ corresponding to the encoding of subtree of *f* can be considerably more succinct compared to the inlining of the entire function bodies in subtree of *f*.

Algorithm 2 creates a PBMC formula for a subtree rooted at *f*, i.e., $\phi _{f}$. The algorithm initially accepts a *substitution scheme* for the function representations and uses it while constructing formula $\phi _{f}$.

#### Definition 3

A substitution scheme for function calls is a function $Sb: F \rightarrow \{inline, sum, nondet\}$ determines how each function call should be handled.

The level of approximation for each function $f \in F$ is determined as one of the following three cases: (i) *inline* when the entire *f* is required to be processed, (ii) *sum* when a pre-computed summary substitutes *f*, and (iii) *nondet* when *f* is treated as a nondeterministic function. Since nondet abstracts away the function, it is equivalent to using a summary formula *true*.

We define three substitution schemes: $Sb_{ inline }\!\!: F \rightarrow \{inline\}$ inlines all the function bodies. $Sb ^{N}_{ eager }\!\!: F \rightarrow \{sum, inline\}$ inlines functions with invalid summaries accumulated in set $N$ and otherwise employs summaries:

4 $$\begin{aligned} Sb ^{N}_{eager} ({g}) =\left\{ \begin{array}{ll} inline, &{}\text{ if }\ g \in N\\ sum, &{}\text{ otherwise } \end{array} \right. \end{aligned}$$

Note that summary mapping $\sigma _{\mathcal {T}}$ is total and all functions initially have summaries. Finally, $Sb ^{N}_{ lazy }\!\!: F \rightarrow \{sum, nondet\}$ treats functions with invalid summaries as nondeterministic calls and the rest as *sum*, as follows:

5 $$\begin{aligned} Sb ^{N}_{lazy} ({g}) =\left\{ \begin{array}{ll} nondet, \quad &{}\text{ if }\ g \in N\\ sum, \quad &{}\text{ otherwise } \end{array} \right. \end{aligned}$$

This results in a smaller initial PBMC formula and leaves the identification of the critical function calls to the refinement loop.

## Motivating example

**Fig. 1:**
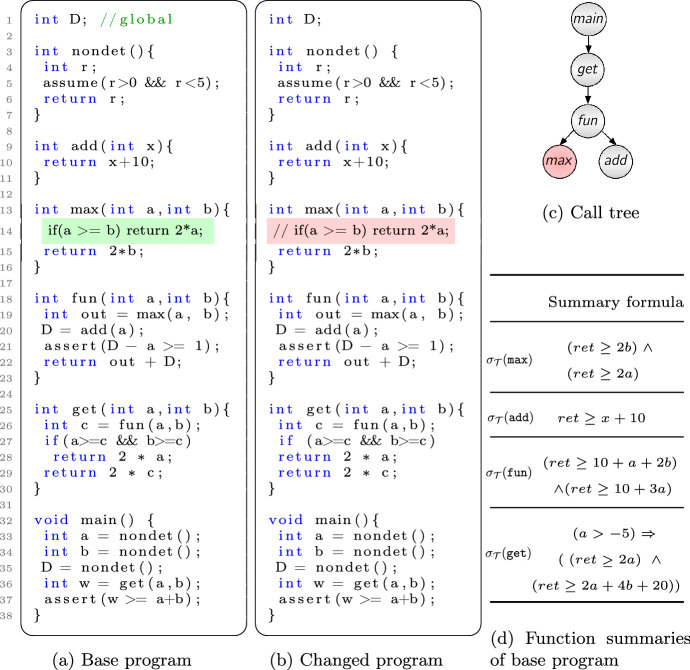
Two versions of a C program with call tree and function summaries

In this section, we demonstrate summary reuse and summary weakening in our incremental verification approach. Consider two programs in Fig. 1 as the base and changed program. We call them $P_1$ (Fig. 1a) and $P_2$ (Fig. 1b). Both versions consist of several functions out of which one function differs, namely $\texttt {max}$, highlighted with red. The function $\texttt {nondet}$ represents a non-deterministic choice (e.g. user input) which is assumed to be in a certain range. The two assert statements capture the property of the program that should always hold after an execution of the program. Program $P_1$ can be encoded as a $\textrm{LRA}$ formula together with the negation of assertions.

Our approach first performs a bootstrapping verification for $P_1$ preferably encoding and solving with a light theory of SMT like $\textrm{LRA}$. After successful verification of $P_1$ using Algorithm 1, summaries of functions are created with respect to all properties, as shown in Fig. 1d. These function summaries represent the relation between the inputs and outputs of each function, and are expressed using a formula that includes the return value of the function, denoted by the variable $ret$ in Fig. 1d. Note that tool implements Algorithm 1 for computing summaries automatically. Summaries are stored for future usage. When it comes to verifying $P_2$, in order to have an efficient verification procedure, instead of performing full-verification again it is desirable to reuse the summaries of $P_1$. We process the changed functions of $P_2$ and investigate if the previous summaries are good enough to over-approximate the changes.

*Summary weakening. * Let us denote $\sigma _{\mathcal {T}}(\texttt {max})$ as summary of $\texttt {max}$ and $\phi ^{'}_{\texttt {max}}$ as $\textrm{LRA}$ encoding of function $\texttt {max}$ in $P_2$. Here the summary check $\phi ^{'}_{\texttt {max}} \Rightarrow \sigma _{\mathcal {T}}(\texttt {max})$ does not succeed. Since $\sigma _{\mathcal {T}}(\texttt {max})$ is conjunctive, we can weaken the summary by dropping some conjuncts to increase the chance of being valid for the changed $\texttt {max}$. As shown in Fig. 1d $\sigma _{\mathcal {T}}(\texttt {max})$ is in conjunctive form:

6 $$\begin{aligned} \sigma _{\mathcal {T}}(\texttt {max}) := (ret \ge 2b ) \wedge (ret \ge 2a). \end{aligned}$$

A possible weakened formula is $\sigma _{\mathcal {T}}^{w}(\texttt {max}):= (ret \ge 2b).$ By dropping a conjunct, the resulting formula is still a summary, but it is coarser than the previous one. Since the implication $\phi ^{'}_{\texttt {max}} \Rightarrow \sigma _{\mathcal {T}}^{w}(\texttt {max})$ is valid, it indicates that the weakened summary is coarse enough to capture the changed function $\phi ^{'}_{\texttt {max}}$. However, the validation check has to propagate towards the caller, i.e., $\texttt {fun}$ to make sure if the coarse summary is a valid over-approximation in the subtree rooted at $\texttt {fun}$.

*Summary reuse. * During the validation of $\texttt {fun}$’s summary, i.e., $\sigma _{\mathcal {T}}$($\texttt {fun}$), if there are summaries available in its subtree they are used. Since function $\texttt {add}$ is not changed, its summary $\sigma _{\mathcal {T}}(\texttt {add})$ is reused straightforwardly as well as $\sigma _{\mathcal {T}}^{w}(\texttt {max})$. However, the summary check for function $\texttt {fun}$ fails because of the change in function $\texttt {max}$, i.e., $\sigma _{\mathcal {T}}(\texttt {add}) \wedge \sigma _{\mathcal {T}}^{w}(\texttt {max}) \wedge \phi _{\texttt {fun}} \Rightarrow \sigma _{\mathcal {T}}(\texttt {fun})$ or $(D\! \ge \! a+10) \wedge (out \! \ge \!2b) \wedge (ret=out+D) \!\Rightarrow \!(ret \!\ge \! 10+a+2b) \wedge (ret\! \ge \! \!10+3a)$ does not succeed. The summary of $\texttt {fun}$ should be weakened to $(ret \ge 10 + a + 2b)$, and the summary check should be performed for function $\texttt {get}$, where the check succeeds. Thus the check does not continue further to the root of the program. This means that the changed program is safe too.

Since there are no further changed functions unprocessed, the incremental checking terminates, and we simply could conclude that $P_2$ is safe too. The weakened summaries ($\sigma _{\mathcal {T}}^{w}(\texttt {max})$ and $\sigma _{\mathcal {T}}^{w}(\texttt {fun})$) are stored instead of the original summaries and will be reused when a new program version arrives.

## Incremental verification of program changes

This section presents our solution to the problem of efficient verification of a program after a change. The problem of incremental verification is stated as follows: given the first program $P_1$ with the safety properties included in the code, a summary mapping (certificate of correctness) $\sigma _{1}$ of $P_1$, and the changed program $P_2$, adapt $\sigma _{1}$ to become a summary mapping of $\sigma _{2}$ for $P_2$, or show that $P_2$ does not admit a correctness certificate (i.e., has a counterexample).

Note that we present the incremental verification algorithm instantiated in the context of BMC and SMT. However, the algorithm is more general and can be applied in other approaches relying on over-approximative function summaries.

### Overview of the algorithm


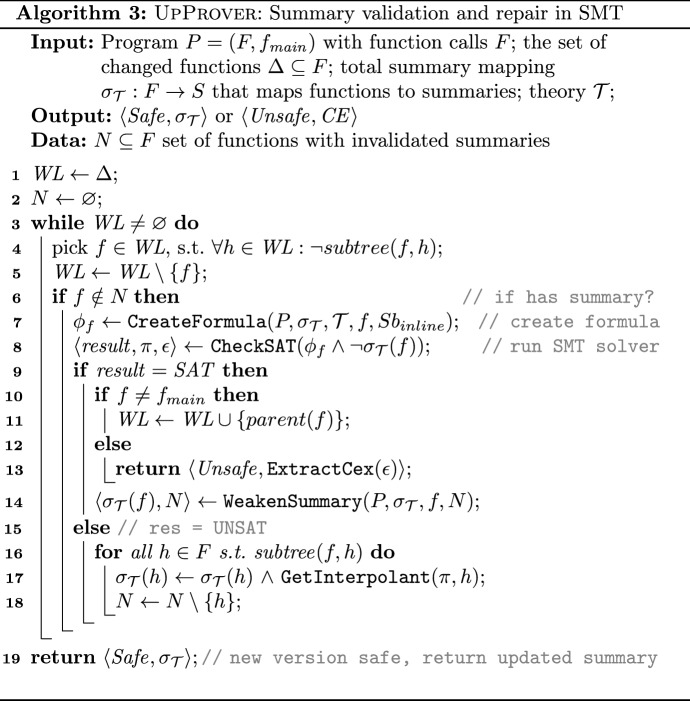


In this section, we first describe the main points of Algorithm 3. Then we describe the important subroutines of the algorithm; (i) an improvement of the algorithm with the summary weakening in Sec. 4.2, and (ii) summary refinement in Sec. 4.3.

Our approach for incremental verification considers two versions of the program, $P_1$ and $P_2$, and the function summaries of $P_1$. If $P_1$ or its function summaries are not available (e.g., at the initial stage), a bootstrapping run (Algorithm 1) is required to verify the whole program $P_2$ to generate the summaries, which are then maintained during the subsequent verification runs. We assume that the set of safety properties remains the same throughout the incremental verification.

As input, the pseudocode takes the new program ($P_2$), the set of functions that have been identified as changed $\Delta$, the theory $\mathcal {T}$, and the summaries as a total mapping $\sigma _{\mathcal {T}}$ from functions *F* to the set of all summaries *S*. Initially in case no summary exists for *f* (e.g., newly introduced in $P_2$) its summary is initialized as *false*. By initializing the summary of *f* as *false*, we are being explicit about the fact that we do not yet have any summary. As output, it reports either $Unsafe$ with a concrete counterexample $CE$, or $Safe$ with a possibly updated total mapping representing the summaries.

The algorithm maintains a worklist $WL$ of function calls that need to be checked against the pre-computed summaries. Initially, $WL$ is populated by a set of functions with code changes, namely $\Delta$ (line 1). Then the algorithm repeatedly chooses *f* from $WL$ so that no function in the subtree of *f* exists in $WL$ (line 4). Then it removes a function *f* from $WL$ and attempts to check the validity of the corresponding summary in the new version. Note that this bottom-up traversal of the call tree ensures that summaries in the subtree of *f* have been already checked (shown either valid or invalid). The algorithm also maintains the set $N$ to store the set of functions with invalid summaries and aims at repairing all of the summaries that were identified as invalid.

The if-condition at line 6 checks whether the summary of *f* is not invalid (i.e., has a summary). If so, $\texttt {CreateFormula}$ constructs the formula $\phi _f$ that encodes the subtree of *f*. Note that here the substitution scenario *inline* is used where all function calls in the subtree of *f* are naively inlined. Later in Sect. 4.3 we introduce a more efficient way for constructing the formula.

The validation check of pre-computed summaries occurs in 8. The validity of implication $$\phi _f \Rightarrow \sigma _{\mathcal {T}}(f)$$ is equivalent to the unsatisfiability of the negation of the formula, $$ \phi _f \wedge \lnot \sigma _{\mathcal {T}}(f)$$. This local formula is sent to an SMT solver for deciding its satisfiability. Performing the local check determines whether the summary is still a valid over-approximation of the new function’s behavior. If the $$ result $$ is $$ UNSAT $$, the validation is successful and the summary covers the changed function. Here, the algorithm obtains a proof of unsatisfiability $$\pi $$ which is used to compute new summaries to update the invalid or missing summaries (line 17). This is called *strengthening* procedure in our approach. If $$ result $$ is $$ SAT $$, the validation of the current summary fails for the changed function (line 9). In this case, either the check is propagated to the function caller towards the root of the call tree (line 11) or a real error is identified (line 13). In the latter case, since the validation fails for the root $$f_ main $$ of the call tree, the algorithm extracts and reports a concrete counterexample from the result of the SMT query (line 13).

Note that if a function *f* is introduced in $P_2$, the caller of *f* is marked as changed by our difference-checker. In such a scenario, since summary of *f* is trivially *false*, the validation check immediately fails, *f* gets added to $N$, and the algorithm continues to check the caller. A successful validation of some ancestor of *f* with inlined *f* generates a summary for *f* (line 17).

Whenever a summary is identified as invalid (line 9), the sub-routine $\texttt {WeakenSummary}$ (line 14) is called to make the summary coarser which is explained in the next section.

### Summary weakening


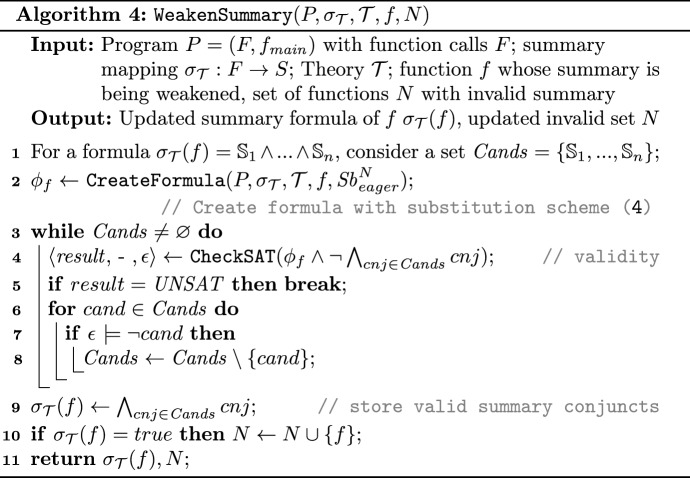


The previous algorithms of incremental verification in [4, 14] check summaries one-by-one and whenever the validation fails, the invalidated summaries are removed straight away, thus the chance to reuse summaries becomes low. In this section instead of removing the invalidated summaries right away, our proposed algorithm attempts to compute coarser over-approximation (i.e. weaker summaries) by dropping some conjuncts, thus maximizing their usability. The key insight behind the algorithm is identifying which parts of the summary break the validity of implication, removing them from the set, and repeating the validation check.

The number of top-level conjuncts in a summary formula is a measure of generalizability of the interpolant. In some applications (see, e.g., [22, 23]) it is useful to further abstract an over-approximation. The idea was inspired by Houdini algorithm [24]. Weakening a summary formula is performed by dropping the conjuncts that break the validity of the summary. Note that Houdini is only meaningful for the summaries with top-level structure in conjunctive form.

In Algorithm 3 once the summary turned out to be invalid (line 9) the sub-routine $\texttt {WeakenSummary}$ (i.e., Algorithm 4) is called to weaken the summaries. Algorithm 4 shows a simple implementation of an iterative check-and-refute cycle that iterates until the validation check of the subset of summary conjuncts succeeds. Initially, summary conjuncts are stored in the set $Cands$ and as the algorithm proceeds, the conjuncts are removed if proven to be invalid.

In line 2 the PBMC formula $${\phi _{f}}$$ for the function *f* is constructed. In this phase $$ Sb ^{N}_{eager}$$ is used as the substitution scheme, i.e., whenever a function summary is available in the subtree of *f*, the summary is used as a substitute for the function body. Then in line 4 the containment of the resulting formula $${\phi _{f}}$$ in the summary candidate is checked by the SMT solver. Once the solver generates a counterexample $$\epsilon $$, it is used to prune the summary conjuncts that break the containment check. This iterates until the solver returns $$ UNSAT $$. In the end, the remaining subset of summary candidates would form a new valid summary for *f*. In case no conjuncts are left, the function summary is assigned to the weakest possible summary, namely *true* (line 10) and it is added to the set $$N$$ that contains functions whose summaries were not valid anymore in $$P_2$$.

Once the weakened summary is obtained from Algorithm 4, in Algorithm 3 the check always propagates to the caller (as the parent is already marked there) to make sure the new weakened summary is suitable in the subtree rooted at the caller. It can be the case that the weakened summary does not capture the whole relevant functionality of the changed function and needs more conjuncts (strengthening) that can be obtained during the validation check of the caller and interpolation in line 17 of Algorithm 3. Note that in this case even though the check propagates to the caller, it would be still beneficial in the sense that the actual encoding of the function body is substituted with the weakened summary, thus the overall check will be less expensive and still maintains some locality.

### Summary refinement


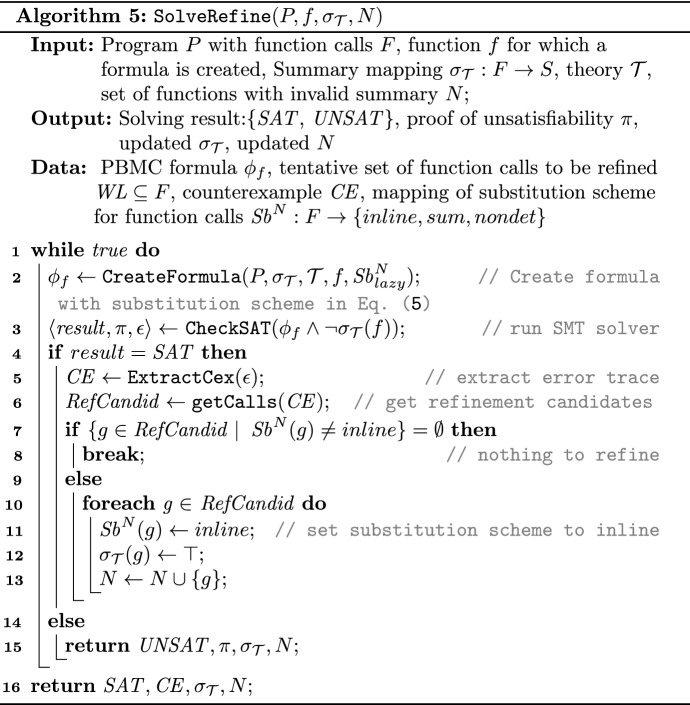


In Algorithm 3 line 7 creates the PBMC formula $${\phi _{f}}$$ in a way that all the nested functions in the subtree rooted at *f* are inlined. In other words, the initial substitution scheme was set to $$ Sb ^{N}_{ inline }$$. To further speed up the incremental check while constructing the formula $$\phi _f$$, pre-computed summaries can be used to abstract away the function calls in its subtree.

In this section we present an algorithm for creating PBMC formulas in a more efficient way, then we present our solution for refining the abstract summaries on demand. The pseudocode in this section can substitute lines 7 and 8 in Algorithm 3.

Algorithm 5 consists of two key points: (i) while constructing the PBMC formula $\phi _f$, whenever summaries are available in the subtree of *f*, they substitute the actual body of the function calls in the subtree, (ii) while checking the validity of summaries, the infeasible behaviors that are detected during analysis of abstract summaries are refined by an iterative refinement procedure.

The initial over-approximation in Algorithm 5 is set to $$ Sb ^{N}_{lazy}$$ where it sets the precision for function calls in the *lazy* style. Then the PBMC formula $$\phi _f$$ is created based on Algorithm 2 with $$ Sb ^{N}_{lazy}$$. The resulting $${\phi _{f}}$$ is expected to be substantially smaller compared to the encoding whose substitution scheme was initially set to $$Sb_{inline}$$.

Then the resulting ${\phi _{f}}$ is checked for the validity whether its pre-computed summary contains the formula, i.e., $\phi _f \Rightarrow \sigma _{\mathcal {T}}(f)$ (line 3). If the resulting formula is satisfiable, it can be either a real or a spurious violation since over-approximative function summaries were used to substitute some of the nested function calls. This can be discovered by analyzing the presence of summaries along an error trace, determined by a satisfying assignment $\epsilon$ returned by a solver and by dependency analysis.

Based on the satisfying assignment the algorithm identifies the set of the summaries used along the counter-example and stores them in $RefCandid$ (line 6). The algorithm applies dependency analysis that restricts $RefCandid$ set to those possibly affecting the validity. Then every over-approximations (summary or nondet) in the $RefCandid$ set is marked as *inline* in the next iteration (line 11). If the set is empty, the check fails and the summary is shown invalid. This refinement loop repeats until the validity of the summary is determined.

## Correctness of the algorithm

This section discusses the correctness of the SMT-based incremental verification algorithm, i.e., given *k* unrolling steps, the algorithm always terminates with the correct answer with respect to *k*. Notice that in this article, program safety is considered with respect to the pre-determined unwinding bound *k*. In the remainder of this section, we assume the same *k* for both old and new programs. In case the user increases the bound for a specific loop, the corresponding function needs to be validated as if changed.

### Correctness of the algorithm

The correctness of Algorithm 3 is stated in the following theorem:

#### Theorem 1

Assume the interpolation algorithm for $\mathcal {T}$ guarantees tree interpolation property. When the Algorithm 3 returns safe, then the entire program is safe, i.e., $error_{f_{main}} \wedge {\varPhi ^{subtree}_{ f_{main} }} \Rightarrow \bot$.

#### Proof

Let $$f_ main $$ be the entry function, $$\sigma _{\mathcal {T}}(f)$$ be the summaries, *f* range over the function calls satisfying $$ subtree (f_ main , f)$$, and $$c_1, \ldots , c_n$$ be the function calls in the (possibly empty) set of functions called by *f*. We first show that the properties

7 $$\begin{aligned}&error_{f_{main}} \wedge \sigma _{\mathcal {T}}(f_ main ) \Rightarrow \bot , \text { and } \end{aligned}$$

8 $$\begin{aligned}&\sigma _{\mathcal {T}}({c}_1) \wedge \ldots \wedge \sigma _{\mathcal {T}}({c}_n) \wedge {\beta _{f}} \Rightarrow \sigma _{\mathcal {T}}(f) \end{aligned}$$

are strong enough to prove that the entire program is safe, and then show that they hold in the algorithm both after successful bootstrapping and after a successful incremental verification run on a set of changes $\Delta$.

*Safety from properties*  (7)  *and*  (8). After rewriting property (7) into $\sigma _{\mathcal {T}}(f_ main ) \Rightarrow ( error_{f_{main}} \Rightarrow \bot )$, logical transitivity and iterative application of property (8) to substitute all interpolants on the right hand side of property (8) yields the inlined formula in the claim $error_{f_{main}} \wedge {\varPhi ^{subtree}_{ f_{main} }} \Rightarrow \bot$.

*Bootstrapping phase.* We show that property (7) holds over the program call tree annotated by computed interpolants whenever bootstrapping verification in Algorithm 1 terminates. Recall that the summaries are generated only when the program is safe with respect to the property, i.e., $error_{f_{main}} \wedge {\varPhi ^{subtree}_{ f_{main} }} \Rightarrow \bot$. Therefore, by definition of interpolation in Definition 1 (property (ii)), $error_{f_{main}} \wedge I_{f_{ main }}$ is unsatisfiable, i.e., property (7) holds. Property (8) follows from our assumption that the interpolation algorithm guarantees the tree interpolation property. This can be seen by choosing the following partitions in Definition 2: $X_i \equiv {\varPhi ^{subtree}_{c_i}}$ for $i \in 1 \dots n$, $Y \equiv {\beta _{f}}$, and $Z \equiv {\varPhi ^{rest}_{f}}$.

*Incremental phase.* Assume that properties (7) and (8) hold before the changes in $\Delta$ are introduced. We show that if Algorithm 3 running on a set of changes $\Delta$ successfully returns $Safe$, both properties are maintained, from which the claim in the theorem follows. If Algorithm 3 successfully terminates, then each function call *c* obtains an updated summary $\sigma _{\mathcal {T}}(c)$ (line 17) when some of its predecessor *f* passed the summary validity check (line 15). Otherwise, the check propagates towards the root of the call tree and eventually may lead to an *UNSAFE* result. Thus, it suffices to show that the recomputed or repaired interpolants satisfy property (8). For this purpose, we again rely on the assumption that the interpolation algorithm guarantees the tree interpolation property. When constructing the formula of a function, Algorithm 2 uses all valid summaries in the subtree of the function. This is sound as we know from property (i) of Definition 1 that $\sigma _{\mathcal {T}}(c_i) \Rightarrow I_{X_i}$ where $I_{X_i}$ is an interpolant obtained from the proof of unsatisfiability corresponding to the successful validity check of the predecessor of $c_i$.

In case some change in $\Delta$ is not contained in pre-computed summary $\sigma _{\mathcal {T}}(f)$ but the algorithm introduced a weakened summary $\sigma _{\mathcal {T}}^{w}(f)$ that contains the change, the algorithm still propagates to the caller function (line 11) to determine whether property (8) holds. In case $\sigma _{\mathcal {T}}^{w}(f)$ is not precise enough in the subtree of the caller, the algorithm proceeds with the refinement of the weak summaries. Once the refined check succeeds, the proof of unsatisfiability is used (through interpolation) to strengthen $\sigma _{\mathcal {T}}^{w}(f)$ (line 17). Technically, the weakened summary and the recomputed summary are conjoined to form a new summary $\sigma _{\mathcal {T}}^{itp }(f) \wedge \sigma _{\mathcal {T}}^{w}(f)$. Again relying on the assumption that the interpolation algorithm guarantees the tree interpolation property, the recomputed and repaired interpolants satisfy property (8). $\square$

### Interpolation algorithms in a concrete theory

We instantiate our generic SMT-based incremental verification approach to certain theories of SMT. In particular, we focus on interpolation algorithms that are the low-level primitives in our approach and discuss the requirements they shall fulfill. The theories of our interest are Linear Real Arithmetic ($\textrm{LRA}$) and Equality Logic and Uninterpreted Functions ($\textrm{EUF}$). Having different theories and interpolation algorithms is of great practical interest since the choice of a good interpolation algorithm may well determine whether an application terminates quickly or diverges.

In the theory of linear arithmetic over the reals, $$\textrm{LRA}$$, there are several efficient proof-based interpolation algorithms proposed in the literature so that the resulting interpolants can differ in ways that have practical importance in their use in incremental verification. For the theory of $$\textrm{LRA}$$, many SMT solvers produce interpolants using application of the Farkas lemma [25]. The most widely used approach computes weighted sum defined by Farkas coefficients of all inequalities appearing in A part of $$(A \mid B)$$ [26]. The interpolant computed in this way is always a *single* inequality. We call this approach Farkas interpolation procedure and denote it as $$ Itp ^{F}$$.

Recently [27] introduced a new algorithm called decomposing Farkas interpolation procedure which is able to compute interpolants in linear arithmetic in the form of a *conjunction of inequalities*. The algorithm is an extension of $$ Itp ^{F}$$; it uses techniques from linear algebra to identify and separate independent components from the interpolant structure. We denote the decomposing interpolation procedure as $$ Itp ^{D}$$. Intuitively, $$ Itp ^{D}$$works as follows: Instead of using the whole weighted sum of *A*, it tries to *decompose* the vector of weights (Farkas coefficients) into several vectors. This effectively decomposes the single sum into several sub-sums. If each of the sub-sum still eliminates all *A*-local variables, the resulting inequalities can be *conjoined* together to yield a valid interpolant.

Since $$ Itp ^{D}$$ is able to produce interpolants in the form of a conjunction of inequalities, it provides the opportunity to make more effective use of summaries in our incremental verification algorithm. Hence, Algorithm 4 can benefit from computing coarser over-approximation (i.e. weaker interpolants) by dropping conjuncts. In the later sections, we will experimentally verify the usefulness of the decomposition scheme by comparing two $$\textrm{LRA}$$ interpolation algorithms.

As for the theory of $\textrm{EUF}$, we use the $\textrm{EUF}$ Interpolation algorithm in  [28] that relies on a congruence [26] graph data structure constructed while solving an $\textrm{EUF}$ problem. $\textrm{EUF}$ interpolation algorithm combines propositional and $\textrm{EUF}$ interpolation which is useful for a model checking setting and some of the conjuncts in $\textrm{EUF}$ interpolants are coming from the propositional structure.

For the propositional part, we use the well-known Pudlák’s interpolation algorithm [29], which is more suitable for function summaries than McMillan’s, since it constructs weaker interpolants and can capture more changes in incremental verification. Note that internally Craig interpolation in propositional logic and theory are combined.

Tree interpolants are used in our incremental algorithm for determining the satisfiability of a first-order logic formula that reuses summaries generated after the unsatisfiability of a slightly different formula is determined. Among the widely used family of interpolation algorithms for $\textrm{LRA}$, $\textrm{EUF}$, and $\textrm{PROP}$ we rely on the ones that can guarantee the tree interpolation property, and thus are suitable for the application in incremental verification of program versions.

The following two lemmas state which $\textrm{LRA}$ interpolation algorithms can guarantee tree interpolation property. The proofs can be found in [20] where we discussed under which conditions $\textrm{LRA}$ interpolation procedures guarantee tree interpolation property.

#### Lemma 1

$\textrm{LRA}$ interpolants computed by Farkas interpolation algorithm have tree interpolation property.

#### Lemma 2

$\textrm{LRA}$ interpolants computed by decomposing Farkas interpolation algorithm with gradual decomposition defined in [20] have tree interpolation property.

The following lemma considers the tree interpolation property of interpolants generated from the same resolution proof in $\textrm{EUF}$, proven in [30].

#### Lemma 3

$\textrm{EUF}$ interpolation algorithm [28] guarantees tree interpolation property.

In a purely propositional setting, we rely on the results from [12, 31] which proves the correctness of the propositional tree interpolation algorithms, stated in the following lemma.

#### Lemma 4

Propositional interpolation algorithm introduced by Pudlák [29] guarantees tree interpolation property.

The proposed solution relies on the above lemmas for the correctness of the incremental verification algorithm in the context of bounded model checking. However, the correctness of the algorithm is not solely restricted to the preservation of the tree interpolation property *by construction*. Instead the correctness of the algorithm can be preserved by checking for the tree interpolation property *on-the-fly*. This means that the algorithm can still produce a correct output even if the tree interpolation property is passed by performing real-time checks in verification run.

It is worth mentioning that our incremental algorithm with theory $\mathcal {T}$ gives the same result as the verification from scratch used in the bootstrapping with the theory $\mathcal {T}$. Although automatically identifying a proper level of encoding is non-trivial (and not a subject of this paper), our approach at least allows various encoding options to the user. In case the algorithm is instantiated with less-precise theory (e.g., $\textrm{EUF}$), if a bug is reported, it might be due to the abstract theory usage, and it is recommended to repeat the verification with a more precise theory (and accordingly, more precise summaries).

## Tool architecture and implementation

**Fig. 2:**
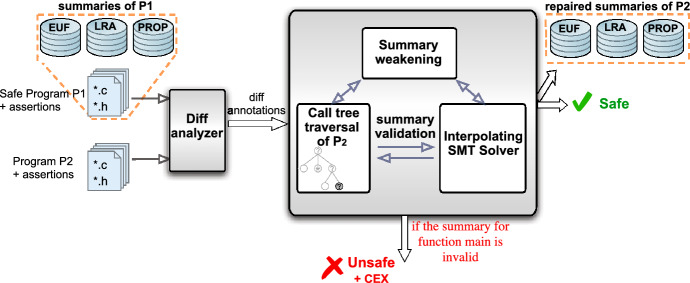
Overview of the UpProver architecture.  UpProver operates at one particular level of precision at each run

This section describes the implementation of our algorithms in the UpProver tool which is a bounded model checker written in C++. UpProver concentrates on incremental verification of program versions written in C. After each successful verification run, it maintains a database of function summaries to store its outputs, which become available as inputs for verification of each subsequent program version. For bootstrapping verification UpProver uses HiFrog standalone bounded model checker [11]. For satisfiability checks and interpolation UpProver uses SMT solver OpenSMT. For pre-processing as a front-end, UpProver uses the framework from Cprover 5.11^2^ to symbolic encoding of C by transforming C program to a monolithic unrolled BMC representation that we use as a basis for producing the final partitioned logical formula.

The architecture of UpProver tool is depicted in Fig. 2. UpProver implements proposed algorithms by maintaining three levels of precision—$\textrm{LRA}$, $\textrm{EUF}$, and purely propositional logic ($\textrm{PROP}$)—to check the validity of pre-computed summaries. The rest of this section describes UpProver’s key components in more detail.

*Difference analyzer*: UpProver performs source code differencing at the level of SSA forms for both the old and the new program to identify a set of functions with code changes. It annotates the lines of code changed between $P_1$ and $P_2$. This defines the scope of summary validations. The user may choose an inexpensive syntax-level difference or a more expensive and precise semantic-level difference that compares programs after some normalization and translation to an intermediate representation [4]. The functions that have been identified as changed are stored in set $\Delta$ in Algorithm 3.

*Call tree traversal*: The call tree traversal guides the check of pre-computed summaries for the modified functions in bottom-up order. It exploits the SMT solver to perform summary validation. When necessary it performs an upwards refinement to identify parent functions to be rechecked using SMT solver or performs summary refinement to refine the imprecise summaries in the subtree.

*Summary repair*: Summaries of $P_1$ (of the selected level of precision) are taken as input and used in the incremental summary validation when necessary. The tool iteratively checks if the summaries are valid for $P_2$ and repairs them on demand, possibly by iterative weakening and then strengthening using interpolation over the refined summaries.

*SMT solving and interpolation engine*: For checking BMC queries and computing interpolants, UpProver interacts with the SMT solver OpenSMT [32]. The solver produces a quantifier-free first-order interpolant as a combination of interpolants from resolution refutations [33], proofs obtained from a run of a congruence closure algorithm in EUF [28], Farkas coefficients obtained from the Simplex algorithm in LRA [27], and Decomposed Farkas interpolation in LRA [27].

*Summary storage*: UpProver takes summaries $\sigma _1$ of $P_1$ as input and outputs summaries $\sigma _2$ of $P_2$. The user defines the precision of $\sigma _1$, and it uniquely determines the precision of $\sigma _2$. In the best-case scenario, the tool validates $\sigma _1$ and copies it to $\sigma _2$. When some of the summaries require repair, the tool produces new interpolants from the successful validity checks of the parent functions and stores them as the corresponding summaries in $\sigma _2$ (while all other summaries are again copied from $\sigma _1$). No summaries are generated when the tool returns $Unsafe$.

## Experimental evaluation

To evaluate our algorithm, we aim to answer the following research questions:

**RQ 1**: Is the use of SMT instead of SAT in incremental verification efficient for real-world programs?
**RQ 2**: Is reusing function summaries beneficial in incremental verification?
**RQ 3**: How does our approach compare with other incremental verifiers?

**Benchmarks and setup:**^3^ We chose 2670 revision pairs of Linux kernel device drivers from [34]. The benchmarks were chosen so that they are parsable by Cprover 5.11, and contain at least one safety property (code assertion). The crafted benchmarks mainly stress-test our algorithm and have changes such as function addition/deletion, signature change, semantic/syntactic change in function-bodies, etc. In addition, we included 240 tricky hand-crafted smaller programs. The crafted benchmarks mainly stress-test our implementation and include function additions/deletions, signature changes, semantic/syntactic changes in function-bodies, etc. On average, the benchmarks have 16’000 LOC, the longest ones reaching almost 71’000 LOC. For each run, we set a memory limit of 10 GB and a CPU time limit of 900 s. The experiments were run on a CentOS 7.5 $\hbox {x}86\_64$ system with two Intel Xeon E5-2650 CPUs, clocked at 2.30 GHz, and 20 (2 x 10) cores. UpProver is available as open-source software. Technical information about the setup of the tool can be found at http://verify.inf.usi.ch/upprover.

**Fig. 3:**
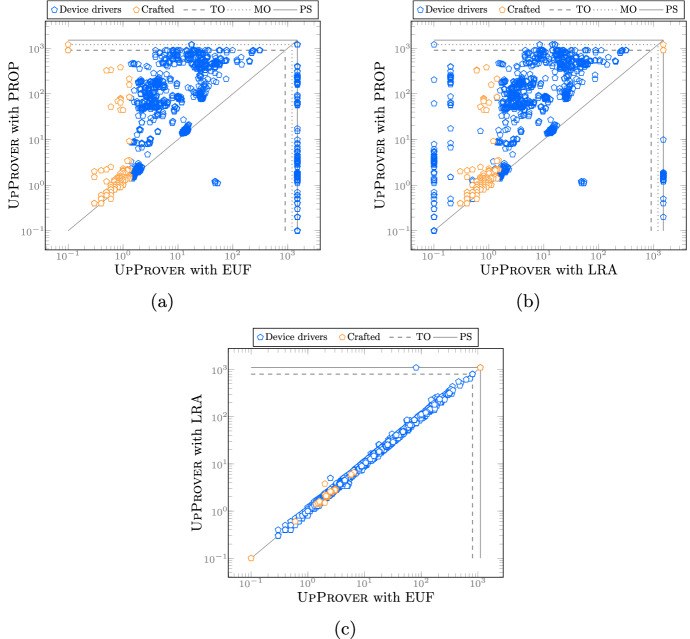
Demonstrating the impact of theory encoding by comparing timings of $\textrm{LRA}$/$\textrm{EUF}$ encodings in UpProver vs. $\textrm{PROP}$ encoding. The inner lines **TO** and **MO** refer to the time and memory limit. The outer lines **PS** refer to the results that are potentially spurious due to the use of abstract theory

### Demonstrating usefulness of different theories

To answer RQ 1, we compare the run time of incremental verification using different encodings. Each point in Fig. 3 corresponds to an incremental verification run of a single benchmark (changed program $P_2$). Figure 3a and b compare the $\textrm{EUF}$/$\textrm{LRA}$-based encodings in UpProver against the $\textrm{PROP}$-based encoding in UpProver 4. Almost universally, whenever run time exceeds one second, it is an order of magnitude faster to verify with $\textrm{LRA}$ and $\textrm{EUF}$ than with $\textrm{PROP}$. In addition, a large number of benchmarks on the top horizontal lines suggests that it is possible to solve many more instances with $\textrm{LRA}$/$\textrm{EUF}$-based encoding than with $\textrm{PROP}$-based encoding. However, the loss of precision is seen on the benchmarks on the vertical line labeled potentially spurious (PS), indicating if the verification result using $\textrm{LRA}$/$\textrm{EUF}$ is unsafe, the result might be spurious because of abstraction. Since UpProver only operates at one particular level of precision at each run, once the tool reports $Unsafe$ in $\textrm{EUF}$/$\textrm{LRA}$ it is recommended to confirm it by a stronger theory encoding.^5^

The results for benchmarks show the trade-off between the precision and run time of incremental verification. In fact the theories are complementary. This can be contrasted to the plot in Fig. 3c where we extracted benchmarks that have successful bootstrapping phase in both $\textrm{LRA}$ and $\textrm{EUF}$ and ended up with 2516 versions out of which 50% are strictly faster in $\textrm{EUF}$ and 30% are strictly faster in $\textrm{LRA}$. The time overhead observed in $\textrm{LRA}$ compared to $\textrm{EUF}$ is due to the more expensive decision procedure.

### Demonstrating the effect of summary reuse

To answer RQ 2 we demonstrate how summary reuse and summary repair benefit incremental verification. To this end we first compare the performance of the tool with and without summary reuse, and then we show how summary repair results in more summaries while the overhead of producing more summaries is negligible.

#### Incremental BMC vs monolithic BMC

**Fig. 4:**
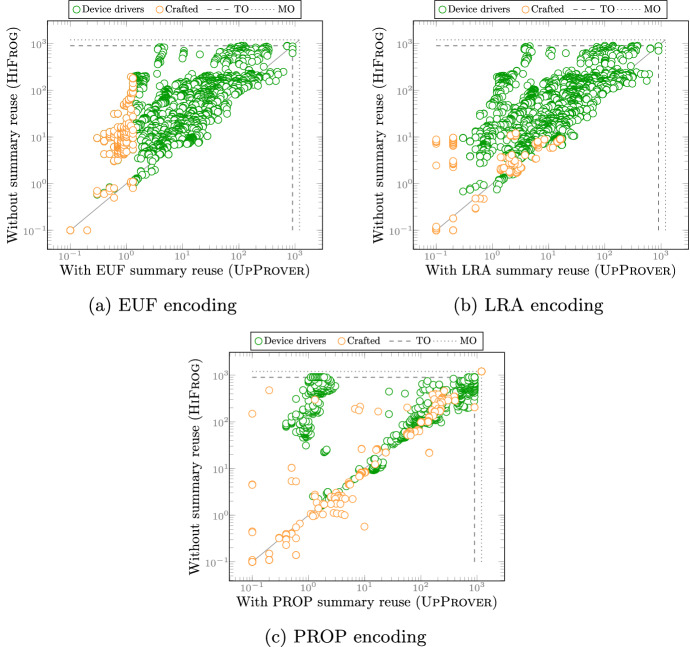
Incremental verification time of UpProver versus non-incremental verification time of HiFrog on (a) EUF, (b) LRA, and (c) PROP encoding

The purpose of this section is to compare verification time of *reusing* the summary against not reusing it. As opposed to summary-based incremental checking in UpProver that maintains and reuses over-approximating summaries of the functions across program versions, a standalone BMC tool, e.g., HiFrog [11] and cbmc [16], creates a monolithic BMC formula and solves it as a standalone run without reusing information from previous runs of other versions. In this section, we compare UpProver with HiFrog as a representative sample of non-incremental BMC tool. This choice is made because both tools use the same infrastructure from Cprover v5.11 to transform C program to obtain a basic unrolled BMC representation that UpProver uses as a basis for producing the final logical formula. Since UpProver and HiFrog share the same parser and the same SMT solver OpenSMT the comparison is not affected by unrelated implementation differences.

The plots in Fig. 4 compare UpProver against HiFrog (non-incremental) with three encodings $\textrm{EUF}$, $\textrm{LRA}$, and $\textrm{PROP}$. Each point in each plot corresponds to verification run of a changed program, with the running time of UpProver when *reusing* the summary of the first version on *x*-axis, and the running time of HiFrog without reuse on *y*-axis. The plots demonstrate the performance gains of incremental verification with reusing function summaries against not reusing it. A large amount of points on the upper triangle lets us conclude that UpProver is an order of magnitude faster than the corresponding non-incremental verification for most benchmarks.

**Table 1:** Number of benchmarks solved by each encoding in UpProver

	EUF		LRA		PROP	
Results	${P_1}$	${P_2}$	${P_1}$	${P_2}$	${P_1}$	${P_2}$
Safe	2529	2514	2686	2663	1249	1186
Unsafe	$268^{{*}}$	$11^{{*}}$	$95^{{*}}$	$20^{{*}}$	31	15
Time out (TO)	113	4	129	3	1536	37
Memory out (MO)	0	0	0	0	94	11
Uniquely verified	–	11	–	41	–	4
No summary ($$no-sum $$)	–	381	–	224	–	1661
Total program versions	2910					

Table 1 provides more details on each encoding that would clarify the scatter plots further. We use acronyms $P_1$ and $P_2$ for two versions of a program. The row $Safe$ indicates the number of programs reported safe by each encoding. In total out of 2910 benchmarks, UpProver with $\textrm{LRA}$ verified safe the largest amount of $P_2$, i.e., 92% and with $\textrm{EUF}$ and $\textrm{PROP}$ verified 85% and 40% respectively. The row $Unsafe$ indicates the number of programs reported unsafe by each encoding. The unsafe results might be spurious when theory encodings were used (indicated by an asterisk). The row TO shows that while UpProver with $\textrm{PROP}$ times out in 53% of the benchmarks, for $\textrm{LRA}$ and $\textrm{EUF}$ this happens for less than 1%. The row MO shows that with $\textrm{PROP}$ encoding, UpProver exceeds the memory limit in 105 benchmarks of $P_1$ and $P_2$, for $\textrm{LRA}$ and $\textrm{EUF}$ this does not occur.

The row *uniquely verified* programs indicates how many $P_2$ can be incrementally verified safe in each encoding exclusively. The count of the uniquely verified using $\textrm{LRA}$ is comparable to other encodings where 41 instances are not solved by any other encoding. Even though, $\textrm{LRA}$ solves the most safe program versions, there are several benchmarks that can be uniquely verified by $\textrm{EUF}$ (11 instances) and by $\textrm{PROP}$ (4 instances). These distinctly verified programs in each encoding can be included in a portfolio.

The row $$no-sum $$ represents the cases where there are no possibility to perform incremental verification because no function summaries were produced in the bootstrapping phase. This can happen when the bootstrapping verification of $$P_1$$ results in $$ Unsafe $$, TO, or MO. The $$\textrm{PROP}$$ encoding results in the highest rate of $$no-sum $$, i.e., 57% (1661) which is the summation of Time Out, Memory Out, and Unsafe results of bootstrapping of $$P_1$$. This asserts that using the rigid approach of bit-blasting for majority of our real-world benchmarks obstructs the incremental verification. On the contrary, $$\textrm{LRA}$$ and $$\textrm{EUF}$$ encodings have a relatively small rate of $$no-sum $$.

It is worth noting that we compared the results of UpProver with the expected results of SVCOMP, since most benchmark names indicate the expected result. Out of 2910 pairs of benchmarks, 2794 pairs had both versions classified as safe. However, for the remaining 116 benchmarks, no expected result was provided and they were marked as unknown, thus we are unable to obtain precise numbers for $Unsafe$ benchmarks. For $Safe$ benchmarks, we never encountered any disagreement with the expected results. This indicates a high degree of accuracy for UpProver in verifying safe benchmarks with theories. However, for $\textrm{EUF}$/$\textrm{LRA}$, $Unsafe$ results might be either real error or false alarms. Due to these unknown benchmarks, we are unable to report the exact number of false alarms for $Unsafe$ benchmarks in theory and we mark them with asterisk.

The overall findings from our experiments show evidence for the following key points: precision and performance-gain present a trade-off. UpProver with $\textrm{EUF}$ and $\textrm{LRA}$ have a better performance compared to the bit precise $\textrm{PROP}$ encoding and are crucial for scalability. At the same time, there is a small number of benchmarks that require $\textrm{PROP}$. Despite the fact that bit-blasted models are more expensive to check than the $\textrm{EUF}$ and $\textrm{LRA}$ models, we find it surprising that the light-weight encodings succeed so often. In practice, the encodings complement each other, and the results imply an approach where the user gradually tries different precisions until one is found that suits the programs at hand.

####  Number of repaired summaries

In this section, we measure the number of repaired summaries that are generated by two out of the box $\textrm{LRA}$ interpolation algorithms. Recall the two phases of the repair in our algorithm: once an existing summary is marked invalid for a changed function *f*, Algorithm 4 first *weakens* the summary by removing broken conjuncts of the summary. In case the weakened summary is not strong enough, Algorithm 3 (line 17) *strengthens* the weakened summary by recomputing interpolants for *f* and conjoining with the weakened summaries. We shortlisted 43 pairs of C programs whose summaries was repaired at least once during incremental verification.

**Fig. 5:**
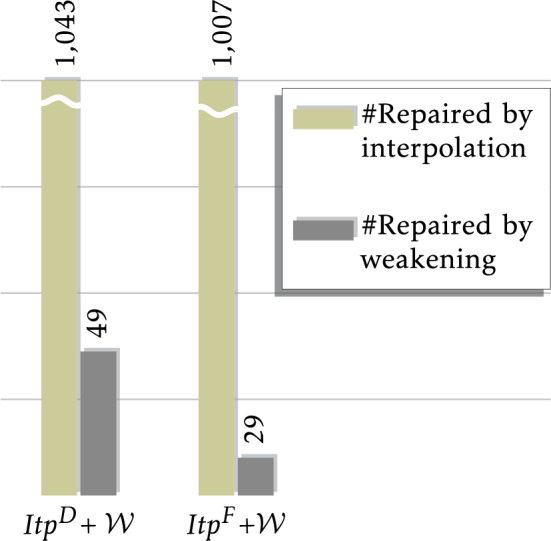
Number of repaired summaries in LRA

Figure 5 depicts the count of two types of repair in $$\textrm{LRA}$$. We use acronyms $$\mathcal {W}$$ for weakening, $$ Itp ^{D}$$ for decomposing Farkas interpolation algorithm, and $$ Itp ^{F}$$ for Farkas interpolation algorithm. We ran Algorithm 1 with $$\textrm{LRA}$$ encoding over the shortlisted programs and generated 3837 $$\textrm{LRA}$$ summaries in total, out of which 1043 summaries were strengthened by $$ Itp ^{D}$$ interpolation and 49 summaries were weakened by $$\mathcal {W}$$. The remaining summaries were either used without any repair, or unused at all due to their corresponding functions did not have change, thus no summary validation performed. Similarly, 1007 and 29 of summaries were repaired by $$ Itp ^{F}$$ and by $$\mathcal {W}$$ respectively.

We can also view the result of this experiment from a different perspective. It can be seen as a way to test how good the interpolants are and how beneficial is the summary weakening. In the summary validation phase in UpProver, the more general interpolants are, the more likely they contain changes of the functions in the new version. Experimenting with $$ Itp ^{D}$$ and $$ Itp ^{F}$$ algorithms for producing $$\textrm{LRA}$$ interpolants, shows that almost always they were as good as they could obtain more summaries with $$\mathcal {W}$$ technique. Observing that 49 summaries out of 3837 could be weakened further, implies that pre-computed interpolants are already as weak as possible but strong enough to be safe.

Now that we have an estimate of the number of function summaries repaired by $\mathcal {W}$, the question becomes how much overhead time this approach adds up to the overall process of UpProver.

#### Overhead of summary repair

**Fig. 6:**
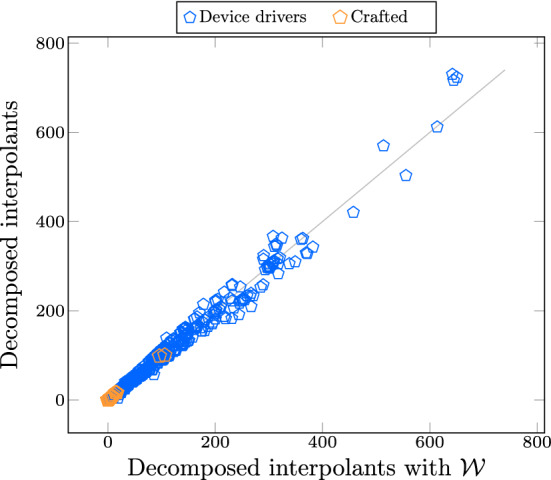
Incremental verification time of UpProver with $\textrm{LRA}$ decomposed interpolants with and without weakening ($\mathcal {W}$)

**Fig. 7:**
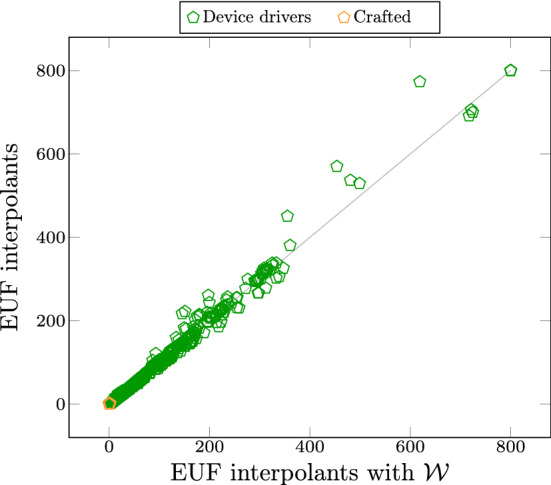
Incremental verification time of UpProver with $\textrm{EUF}$ with and without weakening ($\mathcal {W}$)

In the following, we study the overhead of weakening ($$\mathcal {W}$$) process in the verification time. Figure 6 compares the runtime of UpProver when reusing summaries generated by $$ Itp ^{D}$$with and without $$\mathcal {W}$$^6^. In 60% of the benchmarks $$ Itp ^{D}$$ with $$\mathcal {W}$$ outperform or equal to $$ Itp ^{D}$$. In 32% strictly $$ Itp ^{D}$$ with $$\mathcal {W}$$ faster than $$ Itp ^{D}$$, whereas in 41% $$ Itp ^{D}$$ is strictly faster than $$ Itp ^{D}$$ with $$\mathcal {W}$$. This reveals that in most of the cases not only weakening of summaries did not introduce considerable overhead, but also sometimes outperform the cases without $$\mathcal {W}$$. For instance, the cases that are above 200 s, 29 benchmarks with $$ Itp ^{D}$$ and $$\mathcal {W}$$ strictly outperform $$ Itp ^{D}$$, suggesting that the $$ Itp ^{D}$$ is not undesirable.

Overall, the results imply that summaries repaired by $$\mathcal {W}$$ and strengthened by $$ Itp ^{D}$$ are beneficial in a sense that leads to more summaries in the end, and even shows speed-up in some benchmarks compared to disabling $$\mathcal {W}$$, thus did not affect the overall performance.

In Fig. 7 we compared the runtime of UpProver in theory of $\textrm{EUF}$ with and without $\mathcal {W}$. Concretely, at the area of around 350 to 800 s we observe 6 points above diagonal line confirms out performance of $\mathcal {W}$, whereas 3 points below diagonal shows that pure $\textrm{EUF}$ without $\mathcal {W}$ performs better.

Table 2 gives further details on 14 representative pairs of benchmarks whose change type are substantially different and whose summaries had a chance of being repaired at least once. The table consists of four configurations in $$\textrm{LRA}$$. Each row refers to a pair $$(P_1, P_2 )$$ of programs. The columns highlighted in gray color refers to enabling *summary weakening* feature in the algorithm. The columns highlighted in blue color refers to disabling *summary weakening* feature in which the summaries are repaired only by re-computation through interpolation. The column $$interpolation time $$ shows the time for generating all summaries after successful bootstrapping of $$P_1$$ and $$initial summary $$ the number of function summaries in $$P_1$$ which are non-trivial, i.e., are not simply *true* formula. The column $$preserved $$ refers to the number of functions that stayed the same in $$P_2$$ and $$\Delta $$ to the number of changed functions in $$P_2$$. The column $$diff time $$ shows the time taken by difference-checker to identify changes between $$P_1$$ and $$P_2$$. The column $${ validation \, check }$$ refers to iterative validation checks of summaries for checking the containment of summaries. The columns $${ repaired \, by \, itp }$$ and $${ repaired \, by \,{\mathcal {W}}}$$ indicate the number of newly established summaries by re-computation through interpolation and weakening respectively.

**Table 2:** Detailed verification results for four setups in $\textrm{LRA}$

Bootstrapping			Diff			Farkas			Farkas with $\mathcal {W}$				Decomposed Farkas			Decomposed Farkas with $\mathcal {W}$			
Program pair	Interpolation time(s)	Initial summary #	Preserved function #	Changed $(\Delta )$ #	Diff time (s)	Validation check #	Repaired by itp #	Incremental time (s)	Validation check #	Repaired by $\mathcal {W}$#	Repaired by itp #	Incremental time (s)	Validation check #	Repaired by itp #	Incremental time (s)	Validation check #	Repaired by $\mathcal {W}$#	Repaired by itp #	Incremental time(s)
1	0.1	19	23	1	0.1	2	1	1.8	6	1	0	2.2	2	1	1.8	6	1	0	2
2	0.1	19	22	2	0.1	2	1	1.9	5	1	3	2.3	2	1	1.9	5	1	3	2.2
3	0.1	19	23	1	0.1	2	3	2.8	5	1	4	4	2	3	2.8	5	1	4	3.8
4	0.1	16	18	1	0.1	2	1	3.2	4	1	0	**3**	2	1	3.1	4	1	0	**2**.**9**
5	0.1	5	14	5	0.1	5	2	3	8	1	0	3	5	2	3	8	1	0	**2**.**8**
6	0.1	5	14	5	0.1	5	2	3	8	1	0	3	5	2	3.2	8	1	0	**2**.**7**
7	0.1	4	3	4	0.1	5	4	0.2	5	0	4	0.2	5	4	0.2	7	1	3	0.2
8	3.9	370	947	159	0.2	33	50	42	33	0	50	**41**	33	50	41	34	1	49	**36**
9	1.7	448	1401	665	1.5	72	313	161	72	0	313	**160**	72	311	186	78	1	306	**166**
10	1.4	184	634	39	0.1	21	2	42.7	27	1	0	**39**	21	2	43	27	1	0	43
11	18.8	765	1265	787	0.5	214	268	147	225	2	268	**146**	214	268	144	225	2	268	144
12	3.9	325	763	131	0.2	47	87	114	52	1	88	114	47	87	113	51	1	88	**112**
13	8.4	474	1300	52	0.5	27	64	64	37	1	66	77	27	64	57	37	1	66	86
14	4.4	412	1060	63	0.4	34	53	65	36	1	54	**58**	34	53	59	36	1	54	66

Overall, the numbers in the column ${ validation }\,{ check }$ are higher when $\mathcal {W}$ is used since the algorithm has to iterate more to find a subset of conjuncts in the summary formula. Nevertheless, there are benchmarks, highlighted in bold, that show that the performance improves with the use of $\mathcal {W}$. This happens because the weakened summaries can contain more changes and thus be more suitable for incremental verification. Interestingly, the columns *repaired by itp* and *repaired by*  $\mathcal {W}$show that whenever $\mathcal {W}$ is used, more summary formulas are produced. This is desirable for incremental verification. We see from the experiments that the increase in the number of summary formulas results both directly from weakening the summaries and indirectly because each successful validation check generates new interpolants.

### Comparison of UpProver and CPAchecker 

To answer RQ1, we compare UpProver with a widely-used tool CPAchecker which is able to perform incremental verification by reusing abstraction precisions. It is an orthogonal technique to ours, i.e., it is an unbounded verifier and aims at finding loop invariants. Thus, comparing running times does not make sense since running times in UpProver crucially depend on the chosen bound.^7^ Instead, we focus on comparing the speedups obtained with the two techniques since the change of a bound affects a speedup less.

Here we report the results only on device driver instances which both tools could handle. Out of 250 device drivers categories given in https://www.sosy-lab.org/research/cpa-reuse/predicate.html, we selected 34 categories which are suitable for UpProver.^8^ These categories contain in total 903 verification tasks.

**Fig. 8:**
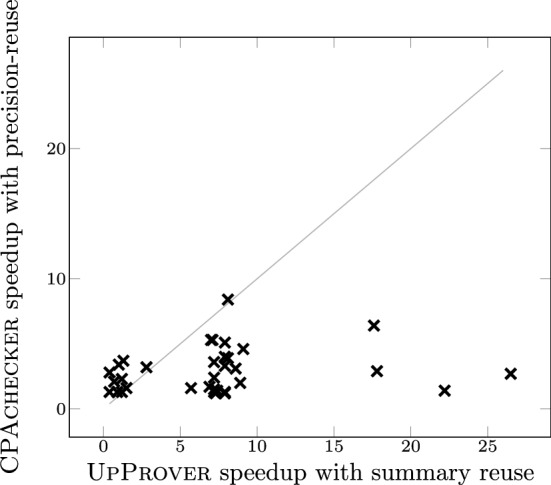
Speedup in UpProver with $\textrm{LRA}$ summary reuse vs. speedup in CPAchecker with precision reuse

Figure 8 shows the comparison of speedup in UpProver and CPAchecker. A large amount of points on the lower triangle reveals that summary reuse in UpProver achieves superior speedup than the precision reuse in CPAchecker. The average speedup in UpProver with $\textrm{LRA}$ summary-reuse is 7.3 with a standard deviation of 6 and in CPAchecker the average speedup is 2.9 with a standard deviation of 1.7. UpProver reported 4 slowdowns among 34 categories, whereas this was not the case for CPAchecker.

## Related work

The problem of incremental verification is not as studied as model checking of standalone programs. There are still several techniques and tools [12, 34–39] which the central incentive behind these lines of work is the ability to reuse intermediate results that were costly computed in previous verification runs, thus achieving performance speedup in the verification of later revisions compared to verification of programs in isolation. Works in this area vary based on the underlying non-incremental verification approach used, which defines what information to be reused and how efficiently so. Various information has been proposed for reuse, including state-space graphs [40], constraint solving results [41], and automata-based trace abstraction [38]. However, these groups of techniques are orthogonal to our approach as we store and reuse the interpolation-based function summaries in the context of BMC for verifying revisions of programs. Moreover, apart from pure reusing the previous computations, our technique repairs the already generated summaries to increase the chance of reusability.

Another approach towards efficiently verifying evolving programs, which is the one we compare in this article, is based on the reuse of previously abstraction precision in predicate abstraction CPAchecker [34]. Apart from the inherent difference that CPAchecker is an unbounded verifier and UpProver is a bounded model checker, we differ from this in that we base our approach on proof-based computing of interpolants and repairing them on the fly, therefore in some sense are able to store more information from the previous runs.

Other techniques for incremental verification of program revisions include reusing inductive invariants in Constrained Horn Clause across programs by guessing syntactically matching variable names [42, 43]. However, these techniques can be applied only for programs sharing the same loop structure. In contrast, our approach is applicable for all sorts of program changes in a bounded model. However, when the changes are drastic, there would not be much summary reuse even with the summary repair.

Other techniques for verifying program versions are based on *relational verification* (also known as regression verification or equivalence checking) which are used to prove equivalence of closely related program versions. To tackle the problem of formally verifying all program revisions various techniques and tools have been proposed for the last two decades [44, 45]. Existing relational verification approaches leverage the similarities between two programs so that they verify the first revision, and then prove that every pair of successive revisions is equivalent [46–50]. Since checking exact equivalence is hard to fulfill and not always practical, there is a group of techniques that check for partial equivalence between pairs of procedures [45, 51, 52] or check conditional equivalence under certain input constraints [46]. Despite the evident success, these techniques are sound but not complete.

The work in [53] investigates the effects of code changes on function summaries used in dynamic test generation. This approach is also known as white-box fuzzing which includes running a program while simultaneously symbolically executing the program to collect constraints on inputs. The aim of [53] is to discover summaries that were affected by the modifications and cannot be reused in the new program version. Since this approach relies on testing, it suffers from the problem of path explosion, i.e., all program paths are not covered. However, this work is orthogonal to our approach as we construct and repair function summaries in a symbolical way, thus our approach allows encoding of all paths of an unrolled program into a single formula.

A group of related work includes techniques using interpolation-based function summaries (such as  [8, 54, 55]) for the standalone programs. Although these do not support program versions, we believe that our incremental algorithm may be instantiated in their context similar to how we instantiated it in the context of HiFrog [11]. The bootstrapping phase of our work is built on top of HiFrog [11], an approach for extracting and reusing interpolation-based function summaries in the context of Bounded Model Checking. In later work [9] we propose to use function summaries more efficiently by lifting function summaries into various SMT levels, thus information obtained from one level of abstraction could be reused at a different level of abstraction.

The work we find most closely related to ours is eVolCheck [4, 12], the predecessor of UpProver, which works only at the propositional level and uses the function summaries only in a bit-precise encoding. Consequently, despite being an incremental approach, eVolCheck is computationally expensive in many cases in practice. Whereas, our approach allows flexibility in balancing between verification performance and precision through both program encoding and the choice of summarization algorithms. As a result of the high-level encodings, UpProver summaries serve as human-readable *certificates of correctness* expressing function specifications.

## Conclusion

We addressed the problem of verifying a large number of programs, in particular, when they are closely related. To avoid expensive full re-verification of each program version and repeating a significant amount of work over and over again, our proposed algorithm operates incrementally by attempting to maximally reuse the results from any previous computations. The key contribution of this work lies in enabling this flexibility by SMT encoding and exploiting the SMT summarization. Having SMT encoding allows for a lot of flexibility when reusing and repairing the summaries leading to the optimization of the whole process which was not possible in the previous SAT-based approach. To achieve incrementality, our algorithm extracts and reuses SMT-based function summaries to over-approximate program functions. It also provides an innovative capability of repairing previously computed summaries by means of iterative weakening and strengthening procedures. Moreover, it offers an efficient way of building formulas and refining them on-the-fly. Through extensive experimentation, we demonstrate that our approach advances the state of the art in incremental verification of program revisions and is significantly more efficient than its predecessor eVolCheck and non-incremental BMC approach.

In future, we plan to extend the tool to handle summaries from different theories simultaneously [9], possibly with the feature of function summarization in theory combination.

## Footnotes

1 For example in [20] this is called the *weak tree-interpolation property.*

2 http://www.cprover.org/

3 The experimentation data including benchmarks, evaluation results, and the source code repositories are available online at http://verify.inf.usi.ch/upprover

4 The $\textrm{PROP}$-based summary reuse in UpProver depicted in Fig. 4c uses the same algorithm from its predecessor eVolCheck but has been significantly optimized compared to its earlier version. Thus $\textrm{PROP}$-based UpProver can be seen as representative of eVolCheck.

5 The work in [9] demonstrated the relation among some theories of SMT from the perspective of over-approximation.

6 The results with $$ Itp ^{F}$$ is similar.

7 For instance, the average running times in CPAchecker is 285.3 s and in UpProver with $\textrm{LRA}$ is 13.4 seconds for chosen bound 5. For other bounds UpProver would have different average running times.

8 The reported version of UpProver is restricted by its dependency on the Cprover 5.11 framework which impedes its frontend from processing some benchmarks.
